# Evaluating the Effects of In Utero Heat Stress on Piglet Physiology and Behavior Following Weaning and Transport

**DOI:** 10.3390/ani9040191

**Published:** 2019-04-24

**Authors:** Christopher J. Byrd, Nichole Chapel Anderson, Drew W. Lugar, Timothy J. Safranski, Matthew C. Lucy, Jay S. Johnson

**Affiliations:** 1 Department of Animal Sciences, Purdue University, West Lafayette, IN 47907, USA; christopher.byrd@ndsu.edu (C.J.B.); chapelnm@missouri.edu (N.C.A.); dwlugar@ilstu.edu (D.W.L.); 2 Department of Animal Sciences, North Dakota State University, Fargo, ND 58108, USA; 3 Division of Animal Sciences, University of Missouri, Columbia, MO 65221, USA; safranskit@missouri.edu (T.J.S.); lucym@missouri.edu (M.C.L.); 4 Department of Agriculture, Illinois State University, Normal, IL 61790, USA; 5 USDA-ARS Livestock Behavior Research Unit, West Lafayette, IN 47907, USA

**Keywords:** behavior, in utero heat stress, piglets, stress physiology, transportation, weaning

## Abstract

**Simple Summary:**

Transporting weaned piglets from the farrowing house to a nursery or grow/finish site is a common but stressful procedure in United States swine production. Piglets removed from the sow at weaning undergo feed and water withdrawal during transportation, and this stress is further compounded by an abrupt change from a liquid to solid diet upon arrival at their destination. As a result, newly weaned and transported piglets exhibit reduced body weight and signs of dehydration post-transport. Unfortunately, in utero stressors can modify the offspring’s physiological, immune, and behavioral responses during postnatal life and may pre-dispose animals to a greater stress response when exposed to novel postnatal procedures, such as weaning and transport. Therefore, the study objective was to evaluate the effects of in utero heat stress on the behavior and physiology of piglets following weaning and transport. We hypothesized that in utero heat-stressed piglets would exhibit a greater behavioral and physiological stress response following weaning and transportation. Additionally, in utero heat-stressed piglets were expected to show physiological signs of metabolic stress immediately following weaning and transport. It was determined that there was some evidence for altered physiological and behavioral responses among in utero heat stressed piglets compared to in utero thermoneutral piglets following weaning and transport.

**Abstract:**

The study objective was to determine whether in utero heat stress (IUHS) affects piglet physiology and behavior following common production practices. A total of 12 gilts were confirmed pregnant and allocated to either heat stress (HS; *n* = 6) or thermoneutral (TN; *n* = 6) conditions on day 30–60 of gestation. At weaning (22.5 ± 2.3 days of age), 1 boar and 1 barrow of median weight were selected from each litter and transported for approximately 7 h. Piglets were then blocked into pens (*n* = 2/pen) by in utero treatment (IUHS (*n* = 12) or in utero thermoneutral (IUTN, *n* = 12)) and sexual status (boar (*n* = 6/in utero treatment) or barrow (*n* = 6/in utero treatment)). Plasma cortisol, non-esterified fatty acids (NEFA), insulin and glucose were evaluated 1 day prior to transport (pre-transport) and immediately after transport (post-transport). Behavioral data were collected on day 1–7 for 60 min at four different time points each day. In utero heat stressed piglets exhibited reduced cortisol concentrations compared to IUTN piglets immediately post-transport (*p* = 0.04). Glucose concentrations were not affected by in utero treatment. Insulin concentrations were reduced in IUTN piglets post-transport compared to pre-transport (*p* = 0.002), but no differences were detected for IUHS pigs. Non-esterified fatty acids tended to be reduced overall for IUHS vs. IUTN pigs (*p* = 0.08). Overall, IUHS piglets performed more drinking behaviors (*p* = 0.02) and tended to perform more aggressive behaviors (*p* = 0.07) than IUTN piglets in the 7 days post-transport. In summary, there was some evidence for altered physiological and behavioral responses among IUHS piglets compared to IUTN piglets following weaning and transport.

## 1. Introduction

Transporting weaned piglets from the farrowing house to a nursery or grow/finish site is a common but stressful procedure in United States swine production [1,2]. Piglets removed from the sow at weaning undergo feed and water withdrawal during transportation, which is further compounded by an abrupt change from a liquid to a solid diet upon arrival at their destination. As a result, newly weaned and transported piglets exhibit reduced body weight and signs of dehydration post-transport [3,4]. In addition, post-transport mixing of unfamiliar piglets increases aggression thereby increasing the likelihood of injury [4,5]. Furthermore, factors such as seasonal temperature variation and the act of loading and unloading may alter behavior and plasma concentrations of stress and immune markers [6,7]. Taken together, these factors may act in an additive manner, placing piglets at a disadvantage for maintaining welfare, growth, and productivity [1], especially if piglets have a pre-existing disorder or altered stress response.

In utero stress can modify the offspring’s physiological, immune, and metabolic response during postnatal life and may pre-dispose animals to a greater stress response and altered behavior when exposed to novel postnatal procedures [8,9]. Previous in utero stress studies with swine have reported altered offspring HPA-axis activity [10,11], lower norepinephrine concentrations, decreased thymus weight, a blunted T-cell and B-cell response to immune challenge [12], and decreased male anogenital distance [13]. Behaviorally, in utero stressed pigs show signs of increased activity [14], perform more escape attempts during open field behavioral tests [15], and alter their response to aggressive interactions [14,16,17]. As a result, in utero stress may exacerbate the severity of the piglet stress response to weaning and transport.

Of the potential in utero stressors, increased environmental temperature is of particular concern for the swine industry, as it is a common, sometimes constant, issue for producers during the summer months and in warmer climates [18]. Additionally, rising global environmental temperatures are expected to continue [19]. Pigs exposed to in utero heat stress (IUHS), defined as exposure of the fetus to maternal body temperatures above euthermic levels, are negatively affected throughout postnatal life [20]. For example, IUHS pigs exhibit a chronically elevated core body temperature [21], decreased head and bone weight as a percentage of body weight [22,23], increased circulating triiodothyronine (T_3_) concentrations and fasting heat production [24], increased fat deposition and circulating insulin concentrations [25], as well as decreased protein accretion [23]. However, little is known about the effects of IUHS in piglets after common production stressors on welfare measures such as the physiological and behavioral stress response.

The study objective was to evaluate the effects of IUHS on the physiology and behavior of piglets following weaning and transport. We hypothesized that IUHS piglets would exhibit a greater physiological and behavioral stress response (increased cortisol; increased aggression and standing, decreased eating, drinking, lying, interaction with straw, and huddling with a pen mate) following weaning and transportation. Additionally, because previous work demonstrated that IUHS pigs have a greater maintenance requirement when compared to IUTN pigs [24], indicators of energy availability and utilization were measured to account for the potential effect of metabolic stress on the physiological and behavioral response to a stressor. We further hypothesized that weaning and transport would lead to a more negative energy balance in IUHS piglets, as compared to their IUTN conspecifics, and this may contribute to reduced welfare.

## 2. Materials and Methods

All experimental procedures were approved by the Animal Care and Use Committees at Purdue University (#1511001332) and the University of Missouri (#8474).

### 2.1. Gestation and Farrowing

Gestation and farrowing procedures were previously described in detail [24,26]. Briefly, 12 first-parity gilts (Landrace × Large White) were bred to the same Duroc sire, confirmed pregnant, and housed in the Brody Environmental Chambers at the University of Missouri (Columbia, MO, USA). Pregnant gilts were maintained in gestation crates throughout all of gestation. From day 30–60 of gestation, 6 pregnant gilts were exposed to thermoneutral (TN; 17.8 ± 0.1 °C; 61.4 ± 0.1% relative humidity (RH)) conditions and 6 pregnant gilts were exposed to cycling heat stress (HS; 28 °C nighttime and 38 °C daytime; 71.1 ± 0.2% RH) conditions [24,26,27]. The period and duration of the heat stress treatment was chosen to reduce the likelihood of pregnancy loss immediately after insemination, and to mimic a late June/early July breeding (i.e., HS during early gestation (mid- to late-summer) and TN during late gestation (early to mid-fall)) to better represent what may actually be occurring in commercial production systems in the midwestern United States. Rectal temperature, skin temperature, and respiration rate were measured in all pregnant gilts at 07:00 and 16:00 h daily. Rectal temperature was measured using a thermistor thermometer (Cole Parmer North America, Vernon Hills, IL, USA), skin temperature was measured on the shoulder of each pregnant gilt using an infrared temperature gun (Raynger ST; Raytek, Santa Cruz, CA, USA) and respiration rate was measured by counting breaths per minute. All thermal measures were greater in HS pregnant gilts compared to TN pregnant gilts from day 30–60 of gestation [24,27]. Following the thermal treatment period (day 61 of gestation) all pregnant gilts were moved to the University of Missouri Swine Teaching farm where they were housed in gestation crates and stayed for the remainder of gestation and subsequent farrowing. While housed at the teaching farm all pregnant gilts were exposed to the same TN conditions (17.8 ± 0.1 °C and 61.4 ± 0.1% RH) until farrowing.

After farrowing, all piglets were housed in TN conditions (26 to 32 °C, [28]). One boar and 1 barrow with body weights near the median of the litter’s range were selected from each litter at weaning (22.5 ± 2.3 days of age; *n* = 2 piglets/litter) for 24 experimental piglets (IUHS, *n* = 6 boars and 6 barrows; IUTN, *n* = 6 boars and 6 barrows). No gilts were used, as the pigs in this study were to be included in a future study on male reproductive physiology.

### 2.2. Transport

One day after weaning, all piglets were transported on 29 December 2015 in an enclosed aluminum trailer from Columbia, Missouri to West Lafayette, IN, USA. Transportation began at approximately 9:00 h and lasted for 7 h and 5 min. A partition inside the enclosed aluminum trailer (1.83 m × 3.66 m) restricted all piglets into a single compartment and lowered the amount of available space to 2.23 m^2^ (0.09 m^2^/piglet), an area allowance within the range recommended by the *Guide for the Care and Use of Agricultural Animals in Research and Teaching* [28]. Straw and wood shavings were placed on the aluminum plated floor for insulation and bedding and to assist with thermoregulation during transit. Feed and water were withheld during transport. During transport, a data logger (Hobo Temperature/RH Data Loggers, Onset Computer Corporation, Bourne, MA, USA) was placed within the trailer to record ambient temperature and relative humidity, but it malfunctioned and data were lost. The average ambient temperature in Columbia, MO, USA on the day of transport was −1.7 °C and the average ambient temperature in West Lafayette, IN, USA was 3.8 °C.

### 2.3. Post-Transportation

Upon arrival, all experimental piglets were housed in 12 pens (*n* = 2 piglets/pen; 1.52 m × 1.83 m) in an environmentally-controlled room in TN conditions (30.2 ± 0.02 °C; 30.1 ± 0.3% RH, [28]), and were blocked by in utero treatment (IUHS, IUTN) and sexual status (boar, barrow). Littermates were not penned together. Plywood was attached along the sides of each pen to remove visual contact between neighboring piglets. Feed and water were provided *ad libitum* via pan feeder and nipple waterer. All diets consisted primarily of corn and soybean meal and were formulated to meet or exceed nutrient requirements [29]. Lighting was set to an automated 12L:12D cycle (L: 6:00–18:00 h). Fresh straw (0.45 kg) was provided at 8:00 h each morning (after removing the previous day’s straw) for the entirety of the study to evaluate differences in interaction with straw between in utero treatment groups.

### 2.4. Blood Analyses

Blood was collected (3 mL; sodium heparin vacutainer tubes; Becton, Dickinson and Company, Franklin Lakes, NJ, USA) 1 day prior to transport (pre-transport) at approximately 1500 h and immediately after transport (post-transport) at approximately 1600 h. Blood was not collected for the remaining 7 day of the study so as not to disturb the natural behavior of the piglets and influence behavioral data collection. Samples were centrifuged (4 °C, 1900× *g* for 15 min), and then plasma was collected, aliquoted, and stored at −80 °C. Commercially available ELISA kits were used according to manufacturer’s instructions to measure plasma cortisol (Cortisol ELISA Kit; Enzo Life Sciences, Inc., Farmingdale, NY, USA) and insulin (Mercodia Porcine Insulin ELISA; Mercodia AB, Uppsala, Sweden) concentrations. A commercially available colorimetric assay was used to measure plasma glucose (Autokit Glucose; Wako Pure Chemical Industries, Ltd.; Chuo-Ku Osaka, Japan) and non-esterified fatty acid (NEFA) concentrations (HR Series NEFA-HR (2); Wako Pure Chemical Industries, Chuo-Ku Osaka, Japan) according to manufacturer’s instructions. The intra-assay and inter-assay coefficient of variation for cortisol, NEFA, glucose, and insulin were 4.2, 2.0, 2.3, 4.8 and 2.4, 2.9, 4.4, 6.5%, respectively.

### 2.5. Post-Transport Behavior

Six video cameras (CT-2M-B2 Bullet Camera; Nuvico Inc., Englewood, NJ, USA) mounted directly above the pens (2 pens/camera) recorded piglet behavior from 6:00–18:00 h on day 1–7 post-transport using a digital video recorder system (GeoVision VMS Software; GeoVision Inc., Tapei, Taiwan). Behavioral data collection from video began at 10:00 h to ensure enough time was available for caretakers to clean pens, provide any needed care, and replace straw each morning. Specific behaviors were quantified individually and included posture (lying, sitting, and standing), feeding, drinking, aggressive interaction, huddling with a pen mate, and interaction with straw (Table 1).

The proportion (expressed as %) of 2-min instantaneous scan samples where pigs were standing, sitting, lying, feeding, and drinking were analyzed in 60 min intervals from 10:00–11:00 h, 12:00–13:00 h, 14:00–15:00 h, and 16:00–17:00 h. The duration of aggressive interactions, huddling with a pen mate, and interaction with straw were collected continuously in 60 min intervals at similar times. All behavioral data were collected by a single trained observer using Observer XT 11.5 behavioral analysis software (Noldus Information Technology B.V., Wageningen, The Netherlands).

### 2.6. Statistical Analysis

All data were analyzed with SAS 9.4 (SAS Institute, Inc., Cary, NC, USA) using a repeated measures design within the GLIMMIX procedure. Concentrations of cortisol, NEFA, insulin, and glucose were included as the dependent variables for models evaluating blood analyses. Individual behaviors were used as the dependent variables for all behavioral models. The proportion of observations (expressed as a percentage of time per day) spent sitting, standing, lying, eating, and drinking, and the percentage of time spent performing huddling, aggression, and straw interaction behaviors on day 1–7 was calculated and averaged by pen. In utero treatment (IUHS, IUTN), blood collection period (pre-transport and post-transport; blood analyses only), day (day 1–7 post-transport; behavioral analyses only), and their interactions were included as fixed independent variables in each model. Sexual status (boar, barrow) was included as a covariate in the model. Sow ID and ELISA plate number were included as random independent variables in models evaluating blood parameters. Assumptions associated with the procedure (homogeneity of variance, normality of error, and linearity) were confirmed prior to running the analyses. Eating behavior data were square root transformed to meet the normality of error assumption and are presented as back-transformed least squares means along with their standard errors obtained via the delta method. All blood analyses are presented as least squares means (±SE). Due to one pen with an uneven number of piglets and one instance of illness during the 7 days post-transport period, only 10 of the 12 total pens were used for posture, eating, and drinking behavioral analyses (IUTN = 4 pens, IUHS = 6 pens; boar = 6 pens, barrow = 4 pens). Additionally, collection of huddling, enrichment interaction, and aggressive behaviors was not reliable for a single IUHS pen due to a small blind spot in the video. Therefore, 4 IUTN pens and 5 IUHS pens (boar = 5 pens, barrow = 4 pens) were used for analysis of these behaviors. A statistical significance between comparisons was defined when *p* ≤ 0.05 and a tendency was defined as 0.05 < *p* ≤ 0.10.

## 3. Results

### 3.1. Blood Analyses

**Figure 1:**
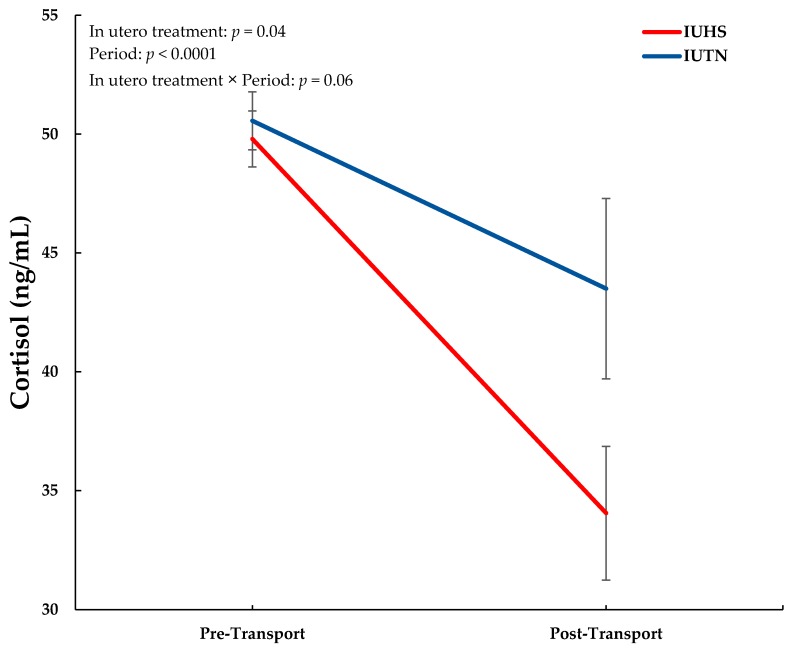
Piglet plasma cortisol concentrations organized by in utero treatment (thermoneutral (IUTN) and in utero heat stress (IUHS)) and period (pre-transport and post-transport).

Overall, IUTN piglets had greater plasma cortisol concentrations compared to IUHS piglets (47.0 ± 2.2 and 41.9 ± 1.8 ng/mL, respectively; F_1,17.58_ = 4.71; *p* = 0.04). This result was largely due to a more pronounced decrease in cortisol concentration for IUHS piglets following transportation (Figure 1). Regardless of in utero treatment, circulating cortisol concentrations were greater during the pre-transport period compared to post-transport (50.2 ± 1.2 and 38.8 ± 2.5 ng/mL, respectively; F_1,16.4_ = 28.5; *p* < 0.0001).

Non-esterified fatty acid concentrations were increased overall post-transport compared to pre-transport (923.5 ± 222.9 and 721.6 ± 225.1 mEq/L, respectively; F_1,18.77_ = 18.77; *p* = 0.009), regardless of in utero treatment. Overall, IUTN piglets tended to have greater circulating NEFA concentrations compared to IUHS piglets (918.0 ± 231.85 and 727.1 ± 222.8 mEq/L, respectively; F_1,13.63_ = 3.36; *p* = 0.09), and this was likely influenced by a more pronounced increase in NEFA concentrations for IUTN piglets post-transport (Table 2).

An in utero treatment by period interaction was detected for circulating insulin concentrations, where IUTN pigs exhibited decreased circulating insulin concentrations post-transport compared to pre-transport (0.022 ± 0.003 and 0.030 ± 0.003 ng/mL, respectively; t_16.0_ = 4.45; *p* = 0.002), but no pre-transport vs. post-transport insulin differences were detected for IUHS piglets (Table 2). No in utero treatment differences were detected (*p* > 0.05; Table 2).

Circulating glucose concentrations were not affected by in utero treatment (*p* > 0.05; Table 2). However, regardless of in utero treatment, circulating glucose concentrations in all piglets decreased post-transport compared to pre-transport (137.4 ± 4.5 and 103.4 ± 1.7 mg/dL, respectively; F_1,17.8_ = 58.5; *p* < 0.0001; Table 2).

### 3.2. Behavior

**Figure 2:**
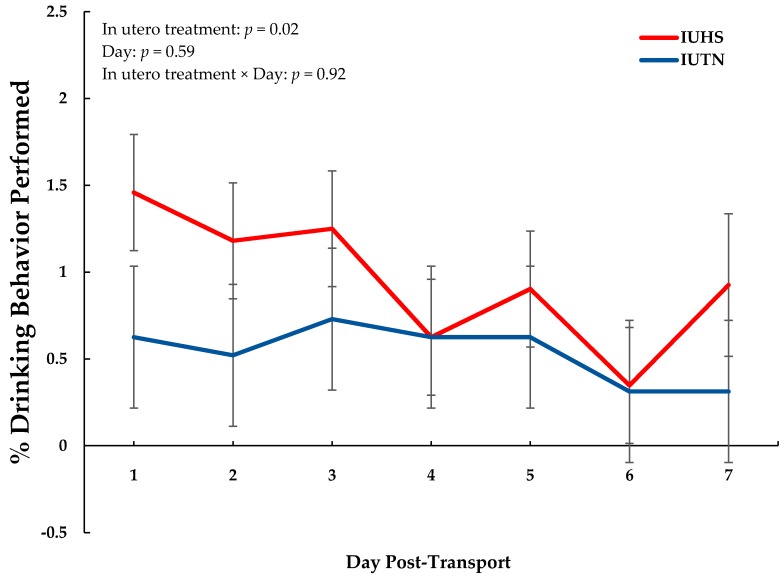
Piglet performance of drinking behavior organized by in utero treatment (thermoneutral (IUTN) and heat stress (IUHS)) over a 7-day period post-transport.

Overall, IUHS piglets tended to perform more aggressive behaviors (F_1,5.2_ = 5.10, *p* = 0.07; Table 3) and performed more drinking behaviors (F_1,6.8_ = 9.96, *p* = 0.02; Table 3; Figure 2) than IUTN piglets. There were no in utero treatment differences detected for posture, huddling, interaction with enrichment, or eating (Table 3).

Sexual status of the piglets affected the performance of aggressive behavior. Overall, boars spent more time performing aggressive behaviors than barrows (1.01 ± 0.11 and 0.28 ± 0.09%, respectively; F_1,4.9_ = 25.2; *p* = 0.004; data not presented).

A day effect was detected for huddling, where piglets, regardless of in utero treatment or sexual status, huddled more on d 3 compared to day 7 (66.0 ± 6.5 and. 34.5 ± 7.2%, respectively; t_41.7_ = 3.31; *p* = 0.03; data not presented). No other behavioral differences or interactions between independent variables were detected (*p* > 0.05).

## 4. Discussion

In utero heat stress has lifelong consequences that can negatively affect pig performance during postnatal life (as reviewed by [20]). However, the impact of IUHS on the postnatal physiological and behavioral stress response of piglets exposed to common production stressors is unknown. Previous studies have determined that in utero stress can affect postnatal offspring behavior and that this postnatal response is associated with increased maternal glucocorticoids that can alter the offspring’s hypothalamic-pituitary-adrenal (HPA) axis during fetal development [30,31]. Therefore, because a marked increase in glucocorticoids, norepinephrine, and epinephrine has been observed in livestock species during times of HS [32,33,34], it is possible that the offspring HPA-axis may be altered due to maternal HS-exposure. Although the mechanism(s) of action are currently unknown in swine, changes in corticosteroid binding globulin availability in the plasma [10,12,35], and altered glucocorticoid receptor expression in the hypothalamus and hippocampus have been described in pigs and may be due to in utero cortisol exposure [10,35]. Furthermore, previous studies have described an increased cortisol response in IUHS pigs exposed to novel postnatal stressors [24]. Despite this however, IUHS piglets in the present study had an overall reduction in circulating cortisol concentrations compared to IUTN piglets, largely due to a more pronounced decrease in cortisol concentrations following weaning and transport. Although this result was unexpected, decreased postnatal cortisol concentrations associated with in utero stress have been previously reported in piglets [10,14,36] and it is possible that IUHS down regulated the HPA-axis feedback set point resulting in decreased postnatal cortisol release in response to a stressor. Alternatively, the decrease in circulating cortisol for IUHS compared to IUTN pigs could indicate that their physiological stress response to weaning and transport was reduced because decreased circulating cortisol levels are a general indicator of reduced stress in pigs [37].

Regardless of in utero treatment, post-transport cortisol levels decreased in all pigs when compared to pre-transport levels. This was unexpected because cortisol generally increases in both young and older pigs following transportation [38,39,40]. However, an explanation for this result may have to do with trip duration, where an increase in cortisol levels may have occurred at the beginning of the transport period and returned to near baseline (i.e., IUTN pigs) or reduced (i.e., IUHS pigs) levels before unloading, as previously described [41]. In the current study, driving time was just over 7 h (not including loading and unloading) with blood collection occurring at weaning and immediately after piglets were removed from the trailer. Therefore, because previous studies focused on swine transport have reported a peak in plasma cortisol levels within 2 h of transportation [38,42], a greater blood collection frequency may be required to detect a transport-induced increase in circulating cortisol and it is possible that cortisol levels had returned to or below baseline at the time of collection in the present study.

In utero heat stress causes a variety of postnatal metabolic changes in pigs (as reviewed by [20]) that may put them at increased risk for distress following weaning and transport. For example, maintenance requirements are increased in IUHS pigs [24], which puts them at an energetic disadvantage compared to IUTN pigs. As a result, this could lead to greater mobilization of energy reserves to meet metabolic demands during times of low energy intake (i.e., weaning and transport) and ultimately cause a decrease in productivity and welfare. Despite this however, in the present study, circulating NEFA concentrations tended to be lower overall in IUHS compared to IUTN piglets. This tendency was likely driven by a greater numerical increase (36%) in post-transport NEFA levels for IUTN pigs. Although these results are contrary to our hypothesis that greater maintenance costs would increase post-transport energy mobilization in IUHS compared to IUTN pigs, they may be explained by differences in insulin concentrations between IUHS and IUTN pigs. During times of low energy intake, glucose levels decrease causing a reduction in circulating insulin and an increase in glucagon levels to promote the mobilization of energy reserves (i.e., NEFAs) for the body [43]. As such, likely due to feed withdrawal during transport, post-transport glucose concentrations were reduced for all pigs compared to pre-transport levels, regardless of in utero treatment. In accordance with this decrease in circulating glucose, post-transport IUTN pig insulin levels were reduced (27%) compared to pre-transport insulin concentrations. However, despite the overall reduction in post-transport circulating glucose, and the fact that IUHS and IUTN pigs were at the same plane of nutrition, no pre-transport vs. post-transport differences in circulating insulin concentrations were detected for IUHS pigs. This maintained elevation in circulating insulin is consistent with previous reports that IUHS causes an increase in postnatal circulating insulin in pigs [25] and likely explains the lack of NEFA mobilization for IUHS pigs in the present study despite the fact that they had not consumed feed for several hours. These data have negative implications towards the ability of IUHS pigs to mobilize energy reserves during periods of feed withdrawal and it is possible that this metabolic stress contributed to the behavioral responses observed in the 7 day post-weaning and transport.

In the current study, IUHS piglets exhibited a 1.8-fold increase in drinking behavior compared to IUTN piglets following weaning and transportation. Because piglets were transported without water and were likely dehydrated [3], it was expected that drinking bouts would be increased initially for all pigs. However, this would not explain the overall maintained increase in drinking behavior of IUHS pigs for 7 day post-transport and could indicate that this behavior is not controlled by normal regulatory mechanisms as previously described [44]. One possible explanation for the increase in drinking behavior may be that the IUHS piglets were performing a stereotypic behavior (i.e., polydipsia is a documented stereotypy in pigs [44,45,46,47]) in response to nutrition-related stress. This is because stereotypic behaviors can appear spontaneously in animals that are feed restricted [44], and it has been documented that feed intake is reduced in piglets during the days following weaning and transport [48] due to a rapid shift from an all milk to all solid diet. As such, polydipsia from feed restriction-induced nutritional stress could have impacted the IUHS piglets more severely due to their aforementioned greater nutritional requirements [24] and inability to mobilize body tissue reserves for energy compared to IUTN pigs. However, while the development of polydipsia as a stereotypy may be one potential explanation, it is important to note that it can take months to develop certain stereotypies in response to stress in pigs [49]. Alternatively, the greater drinking behavior observed in IUHS pigs may be due to drinker manipulation performed during a state of nutritional (food restriction) or environmental (insufficient rooting material) frustration [50] instead of a stereotypic response. Therefore, these data should be interpreted with caution until more research can be conducted to validate the results.

In utero heat-stressed piglets tended to have a 1.6-fold increase in the overall performance of aggressive behaviors compared to IUTN pigs. While increased aggression after transport is common and often continues until a social hierarchy has been established [4,5], greater aggressive behavior may pre-dispose IUHS pigs to fighting and a higher risk of injury due to mixing and transportation. Although the physiological mechanism for why IUHS piglets may be more aggressive than IUTN piglets after transport is unknown, others have hypothesized that the postnatal performance of social behaviors, such as aggression, are affected by alterations to the HPA axis feedback set-point during fetal development [17,51]. This is because the HPA-axis is one of the major hormonal systems underlying the ‘normal stress response’ and mood disorders are often attributed to altered HPA-axis activity [52]. However, attempts to elucidate a potential mechanism for the relationship between HPA-axis function and aggressive behavior in piglets are inconsistent [11,14,17,53,54]. Therefore, more work should be conducted to determine a mechanism for the relationship between HPA-axis function and aggressive behavior in response to IUHS.

While aggressive behaviors only tended to be related to in utero treatment, the performance of aggressive behaviors was greater in boars, as compared with barrows. Although increased aggression commonly occurs in groups of mature boars [55], greater aggression in the present study was unexpected since boars had not reached sexual maturity (6–8 months of age; [56]). Despite this, increased activity and expression of aggressive and mounting behavior has been observed in young boars [57,58], possibly due to an increase in testosterone shortly after weaning (4 weeks of age; [59]). Therefore, greater aggression in boars in the present study may be due to higher testosterone production compared to barrows between 22 and 28 days of age. However, because boar behavior (in the absence of an in utero treatment interaction) was not a primary focus of the study, no attempt to measure testosterone was made and this hypothesis would need to be confirmed in subsequent experiments.

Although this study has provided new insight into the effects of IUHS on the postnatal response of piglets to weaning and transport stress, some limitations should be mentioned. Given the relatively small sample size used in the current study for the final behavioral analysis, the behavioral results should be regarded as preliminary and should be confirmed in future studies that utilize a larger number of piglets to evaluate behavior in response to IUHS. In addition, future studies should include both male and female piglets in order to differentiate between possible sex differences. Despite these potential caveats, the results presented here serve as an important starting point for understanding the effects of IUHS on piglet stress response, metabolic response, and behavior following exposure to common stressors experienced on-farm.

## 5. Conclusions

Stress during in utero development can alter the behavioral and physiological response of animals to postnatal stressors. Therefore, we hypothesized that IUHS would increase the physiological stress response and alter behavior in newly weaned and transported piglets. It was observed that IUHS piglets may perform more aggressive and stereotypic behaviors than IUTN piglets and that IUHS piglets had a reduction in postweaning and transport circulating cortisol compared to IUTN piglets. Furthermore, IUHS piglets were unable to effectively mobilize energy reserves during weaning and transport-induced feed withdrawal. Given the small sample size for behavioral data in the current study, these results should be regarded as preliminary. Future work should incorporate a larger sample size in order to confirm these findings and investigate the long-term impact of altered behavior and physiology following weaning and transport on swine welfare.

## Figures and Tables

**Table 1:** Ethogram used for behavioral data collection.

Behavior	Description
Standing	Piglet is upright with all legs extended perpendicular to the ground. All feet are in contact with the ground.
Sitting	Piglet is upright with front legs extended perpendicular to the ground. Rear legs are not extended. All four feet are in contact with the ground.
Lying	Piglet is sternal or lateral on the ground. Legs may be extended but are parallel to the ground. Feet may be in contact with the ground but do not support the weight of the piglet.
Huddling	Both piglets are lying approximately parallel with at least ¾ body length in contact with one another.
Eating	Piglet is standing at feeder, ingesting feed.
Drinking	Piglet is standing at nipple drinker, ingesting water.
Aggression	Classified as one of three criteria, all resulting in retreat of the other pen mate: (1) Piglet uses head to forcefully push or knock pen mate (non-belly nosing). (2) Piglet mounts (two hind-feet remain on the ground) pen mate. (3) Piglet forcefully bites ears or face of pen mate.
Interaction with enrichment (straw)	Piglet is using one or two feet to scratch, move, or manipulate straw ^1^.

^1^ Rooting was not included in this definition, as the cameras used to monitor behavior were not reliable for distinguishing rooting from other oral behaviors occurring in proximity to the ground.

**Table 2:** Least squares means of circulating concentrations of NEFA (non-esterified fatty acids), insulin and glucose.

Parameter	Pre-Transport		SE	Post-Transport		SE	*p*-Value		
	IUTN	IUHS		IUTN	IUHS		Trt ^1^	Period ^2^	Trt × Period ^3^
NEFA (mEq/L)	777.4	665.7	232.7	1058.7	788.4	231.9	0.08	0.009	0.25
Insulin (ng/mL)	0.030 ^a^	0.023	0.003	0.022 ^b^	0.023	0.003	0.48	0.002	0.003
Glucose (mg/dL)	144.3	130.5	6.3	112.7	102.0	6.6	0.17	<0.001	0.70

IUTN: in utero thermoneutral; IUHS: in utero heat stress; SE: average standard error across treatments for each blood collection period. ^1^ Effect of in utero treatment (IUTN/IUHS) on parameter. ^2^ Effect of period (pre-transport, post-transport) on parameter. ^3^ Effect of interaction between in utero treatment and period on parameter. ^a,b^ Different superscripts indicate a difference of *p* ≤ 0.05 between pre- and post-transport values.

**Table 3:** The effect of in utero treatment on offspring performance (%) of analyzed behaviors.

Behavior	IUTN ^1^	IUHS ^2^	SE ^3^	*P*-Value
Standing	33.80	29.70	5.20	0.59
Lying	65.80	69.70	5.10	0.60
Huddling	46.20	52.10	5.60	0.48
Eating ^1^	5.12	4.31	2.60	0.83
Drinking	0.54	0.96	0.09	0.02
Aggression	0.48	0.80	0.1	0.07
Interaction with straw	1.06	0.71	0.17	0.18

IUTN: in utero thermoneutral; IUHS: in utero heat stress; SE: average standard error across in utero treatments for each behavior. ^1^ Data were square root transformed for statistical analysis. Values presented in the table are back-transformed least squares means ± their standard errors obtained via the delta method.
